# CD8 T cells induce the peritubular capillary rarefaction during AKI to CKD transition

**DOI:** 10.7150/ijbs.96812

**Published:** 2024-05-19

**Authors:** Wei Jiang, Tao-Tao Tang, Yi-Lin Zhang, Zuo-Lin Li, Yi Wen, Qin Yang, Yu-Qi Fu, Jing Song, Qiu-Li Wu, Min Wu, Bin Wang, Bi-Cheng Liu, Lin-Li Lv

**Affiliations:** Institute of Nephrology, Zhong Da Hospital, Southeast University School of Medicine, Nanjing, China.

**Keywords:** CD8 T cells, apoptosis, capillary rarefaction, AKI, CKD, renal fibrosis

## Abstract

Acute kidney injury (AKI) transformed to chronic kidney disease (CKD) is a critical clinical issue characterized by tubulointerstitial inflammation (TII) and fibrosis. However, the exact mechanism remains largely unclear. In this study, we used single-cell RNA sequencing (scRNA-seq) to obtain a high-resolution profile of T cells in AKI to CKD transition with a mice model of unilateral ischemia-reperfusion injury (uIRI). We found that T cells accumulated increasingly with the progression of AKI to CKD, which was categorized into 9 clusters. A notably increased proportion of CD8 T cells via self-proliferation occurred in the early stage of AKI was identified. Further study revealed that the CD8 T cells were recruited through CXCL16-CXCR6 pathway mediated by macrophages. Notably, CD8 T cells induced endothelial cell apoptosis via Fas ligand-Fas signaling. Consistently, increased CD8 T cell infiltration accompanied with peritubular capillaries (PTCs) rarefaction was observed in uIRI mice. More impressively, the loss of PTCs and renal fibrosis was remarkably ameliorated after the elimination of CD8 T cells. In summary, our study provides a novel insight into the role of CD8 T cells in the transition from AKI to CKD via induction of PTCs rarefaction, which could suggest a promising therapeutic target for AKI.

## Introduction

Acute kidney injury (AKI) is a critical condition that is strongly related to elevated risks for the progression of chronic kidney disease (CKD) and end-stage renal disease (ESRD) 1,2. Understanding the transition process from AKI to CKD may provide an important target for developing effective therapies to promote renal repair. While tubulointerstitial inflammation (TII) is widely accepted as a significant factor in driving this process 3,4, the underlying mechanisms responsible for the development of TII are still largely unknown.

TII is primarily initiated through innate immunity response, among which macrophages and dendritic cells (DCs) are regarded as the major immune cells 5,6. The infiltrated innate immune cells consequently recruit the adaptive immune cells, including T and B cells, via various cytokines and chemokines 7,8. Unlike traditional awareness of adaptive immunity as late responders during inflammation, it is increasingly recognized that T cells infiltrate into kidneys at the early stage of AKI 9. T cells are increasingly recognized to be involved in tissue injury and exacerbation of the inflammatory response 10. However, the dynamic landscape of T-cell infiltration and their contributions to the chronic transition of AKI remain largely unclear.

It is well recognized that T cells encompass a variety of subtypes, each with distinct functions. Recent advances have indicated that TH1 and TH17 cells promote innate renal inflammation and injury, and Treg promotes protection and repair 11-13. Upon activation, naive CD8 T cells could mainly differentiate into effector or memory CD8 T cells. Effector CD8 T cells, also referred to as cytotoxic T lymphocytes (CTLs), can induce target cell death directly. Memory CD8 T cells are critical for long-term immunity by providing effective protection upon antigen reencounter 14,15. However, the role of CD8 T cells is still unclear after AKI. Previous studies have revealed that CD8 knockout mice were protected from AKI induced by cisplatin, while its protective role was not apparent in other models 16,17. The implication of CD8 T cells and their interactions with resident renal cells may be distinctive under different circumstances and eventually contribute to divergent disease outcomes. Previous studies have revealed that CD8 T cells are crucial in exacerbating glomerular-related diseases by inducing the injury of podocytes 18,19. On the other hand, PD-L1 partially protects renal tubular epithelial cells from the attack of CTLs *in vitro* 20. However, the interactions between CD8 T cells and renal parenchymal cells in AKI to CKD are poorly unknown and deserve further investigation.

In this study, we demonstrated that CD8 T cells displayed close interaction with endothelial cells (EC) via the Fas ligand (Fasl)-Fas signaling pathway, leading to the apoptosis of EC and rarefaction of peritubular capillaries (PTCs) and renal fibrosis, which was largely reversed by the elimination of the CD8 T cells. Our study identified a novel insight into the role of CD8 T cells in promoting peritubular capillary rarefaction during the AKI to CKD transition, which may provide a new therapeutic target for this critical disease.

## Results

### Single-cell RNA-sequencing atlas of T cells during AKI to CKD transition

**Figure 1:**
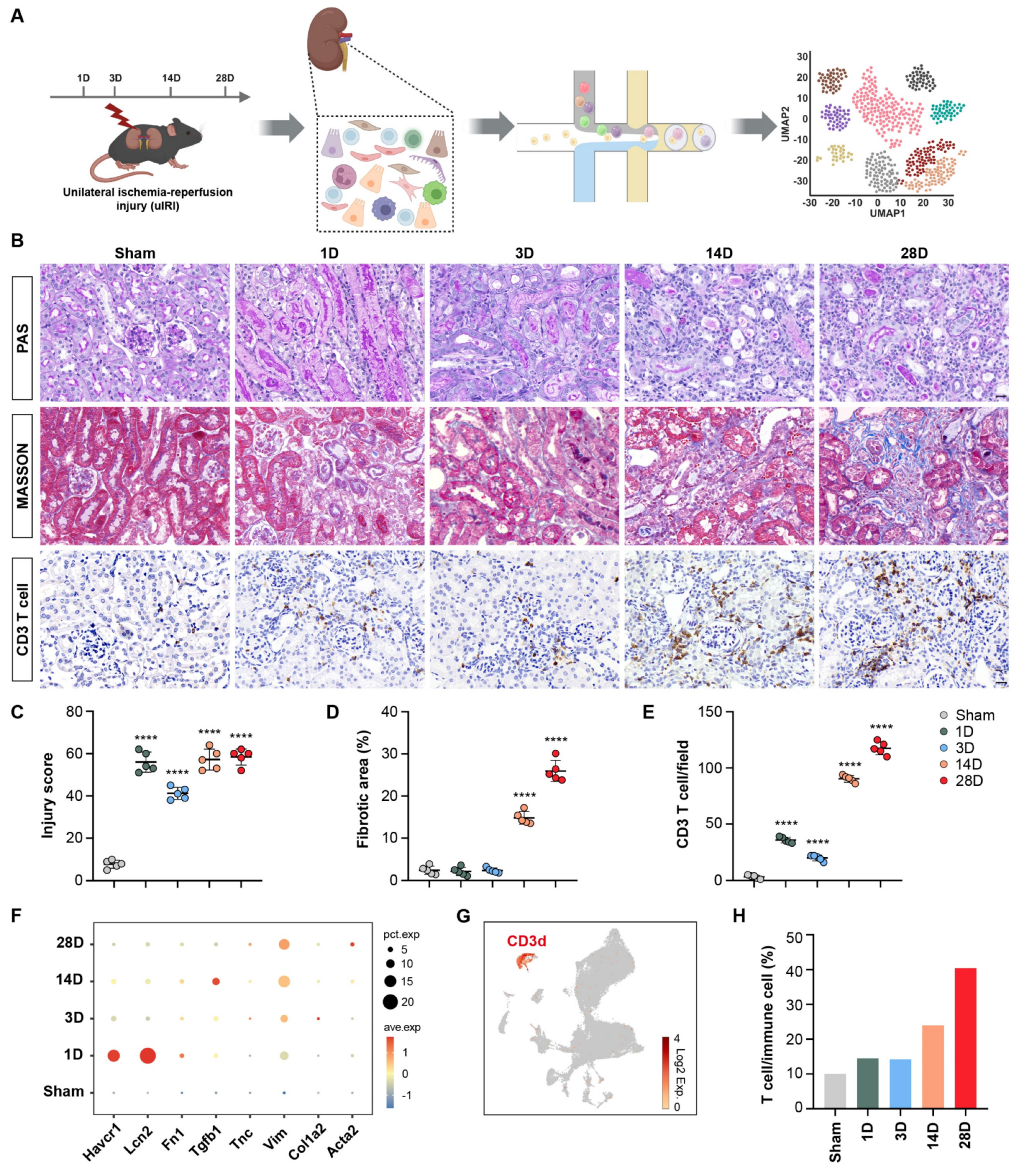
** Single-cell RNA-sequencing atlas of T cells during AKI to CKD transition.** (A) Flow chart illustrated the progress of the experimental project for scRNA-seq analysis. (B) Representative histopathology images of PAS and Masson's staining at each time point in the uIRI model after AKI. Representative images of CD3 immunohistochemical staining at each time point in the uIRI model after AKI. (C-E) Quantification of injury score, fibrotic area, and CD3^+^ T cells based on five mice. (F) Expression levels of Hepatitis A virus cellular receptor 1(Havcr1), Lipocalin 2 (Lcn2), Fibronectin 1 (Fn1), Transforming growth factor beta 1 (Tgfb1), Tenascin C (Tnc), Vimentin (Vim), Collagen type I alpha 2 (Col1a2), and Actin alpha 2 (Acta2) in the kidney after AKI. (G) UMAP plot colored by CD3d, representing T-cell annotation. (H) Proportions of T cells among immune cells after AKI. Scale bars, 20μm. Data are presented as means ± SD. **** *P* < 0.0001 compared to the sham group.

Mice were euthanized 1, 3, 14, and 28 days(D) after operation. The kidneys were then harvested for scRNA-seq analysis, as illustrated in Figure 1A. PAS histologic analysis showed destruction of tubular structure and formation of tubular casts after AKI, while tubular necrosis and continuously increased infiltration of interstitial inflammatory cells, especially T cells accompanied by accumulation of collagenous fibers at chronic stages of 14 and 28D were observed (Figures 1B-1E). Correspondingly, the expression of Havcr1 and Lcn2 increased rapidly after AKI, whereas transforming growth factor beta 1 (Tgfb1), vimentin (Vim), and actin alpha 2, smooth muscle (Acta2) significantly elevated at late stages after AKI, confirming the successful construction of the AKI to CKD transition model (Figure 1F).

Following quality control of scRNA-seq data, 60,010 cells were obtained from the kidneys of both healthy and uIRI mouse models. A UMAP plot was generated to visualize the distribution of CD3d^+^ T cells (Figure 1G). Interestingly, there was a significantly increased proportion of T cells (from nearly 10% to 40%) among immune cells (including Macrophages, Neutrophils, B cells, NK cells, and T cells.) in the chronic stage following AKI (Figure 1H). The composition and function of these T cell populations deserve further investigation.

### The heterogeneity and function of T cell clusters during AKI to CKD transition

**Figure 2:**
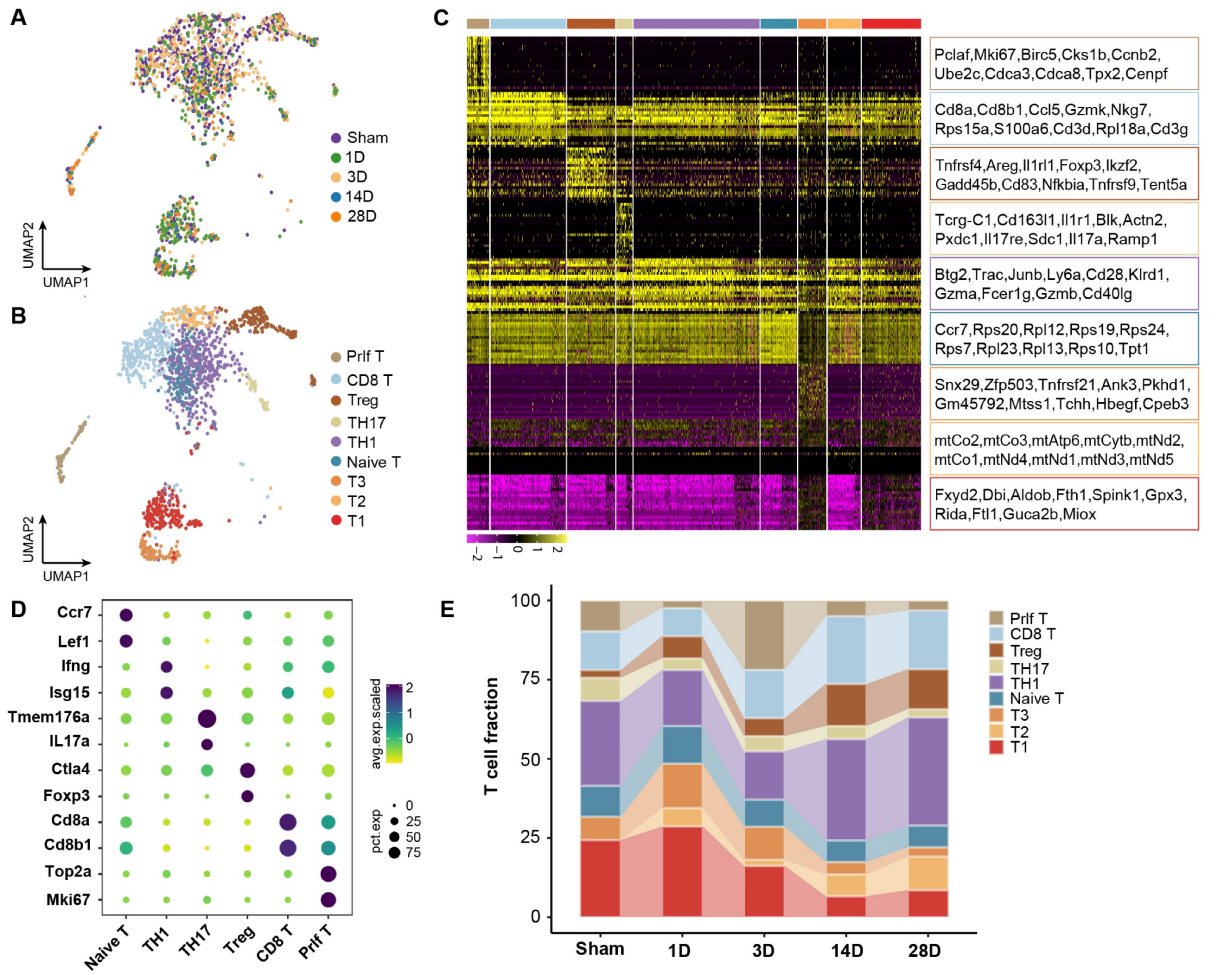
** The heterogeneity and function of T cell subtypes during AKI to CKD transition.** (A) UMAP plot color-coded by T cells at each time point. (B) UMAP plot color-coded by 9 clusters representing T cell annotation. (C) The distribution of the top 20 genes among each T cell subtype in the kidney. (D)Representative marker genes for each T cell subtype in the kidney. (E) The relative contribution of each T cell subtype at each time point.

To investigate the role of T cell clusters during the chronic transition of AKI, UMAP analysis was performed on T cells. Nine distinct clusters were identified with specific transcriptome profiles (Figures 2A and 2B). By comparing to the immune cell bulk RNA-Seq data, the clusters were defined as TH1 cluster (Ifng, Isg15), TH17 cluster (IL17A, Tmem176a), Treg cluster (Foxp3, Ctla4), naive-T cluster (Ccr7, Lef1), Proliferating T cluster (Top2a, Mki67), and CD8 T cluster (CD8a, CD8b1) (Figures 2C and 2D). The remaining three clusters did not exhibit any obvious marker genes for assignment. Interestingly, CD8a and CD8b1 as characteristic genes of the CD8 T cluster were also identified in the proliferating T cluster, indicating a strong connection between these two clusters. Impressively, we identified that CD8, Treg, and Th1 clusters exhibited significantly increased infiltration during the chronic stages, which suggested a significant contribution to the chronic transition of AKI. Notably, a remarkable proportion of the proliferating T cluster on Day 3 was observed, which decreased on 14 and 28D (Figure 2E). The trajectory of this population deserves further analysis.

To elucidate the functions of those T cell clusters, Gene Ontology (GO) analysis was performed. The TH1 and TH17 cluster was enriched in the positive regulation of interferon-gamma and the inflammatory response pathway, which aligned with the previous understanding of those cells (Figures S1A and S1B). Furthermore, the naive and proliferating T cluster enriched cytoplasmic translation and cell cycle-related pathways, indicating a transitional immature cell stage (Figures S1C and S1D). The enrichment of the apoptosis pathway, including the Fas Ligand (Fasl) gene in the CD8 T cluster was observed, suggesting its mature cytotoxic function (Figures S1E; Table S1). Treg was previously identified with potent protective role in AKI, while Treg displayed an accumulation of inflammation-related pathways in our mice model of AKI to CKD transition, which was in line with a recent sc-RNA seq of mouse fibrosis model after AKI (Figures S1F) 9. Thus, we elucidated the dynamic landscape and diverse functions of T cell clusters during the transition from AKI to CKD. CD8 T cluster is characterized by remarkable pro-apoptosis function, which may originate from the proliferating T cluster that occurred at the early stage after AKI.

### Proliferative T cluster differentiates into CD8 T cluster with cytotoxic function

**Figure 3:**
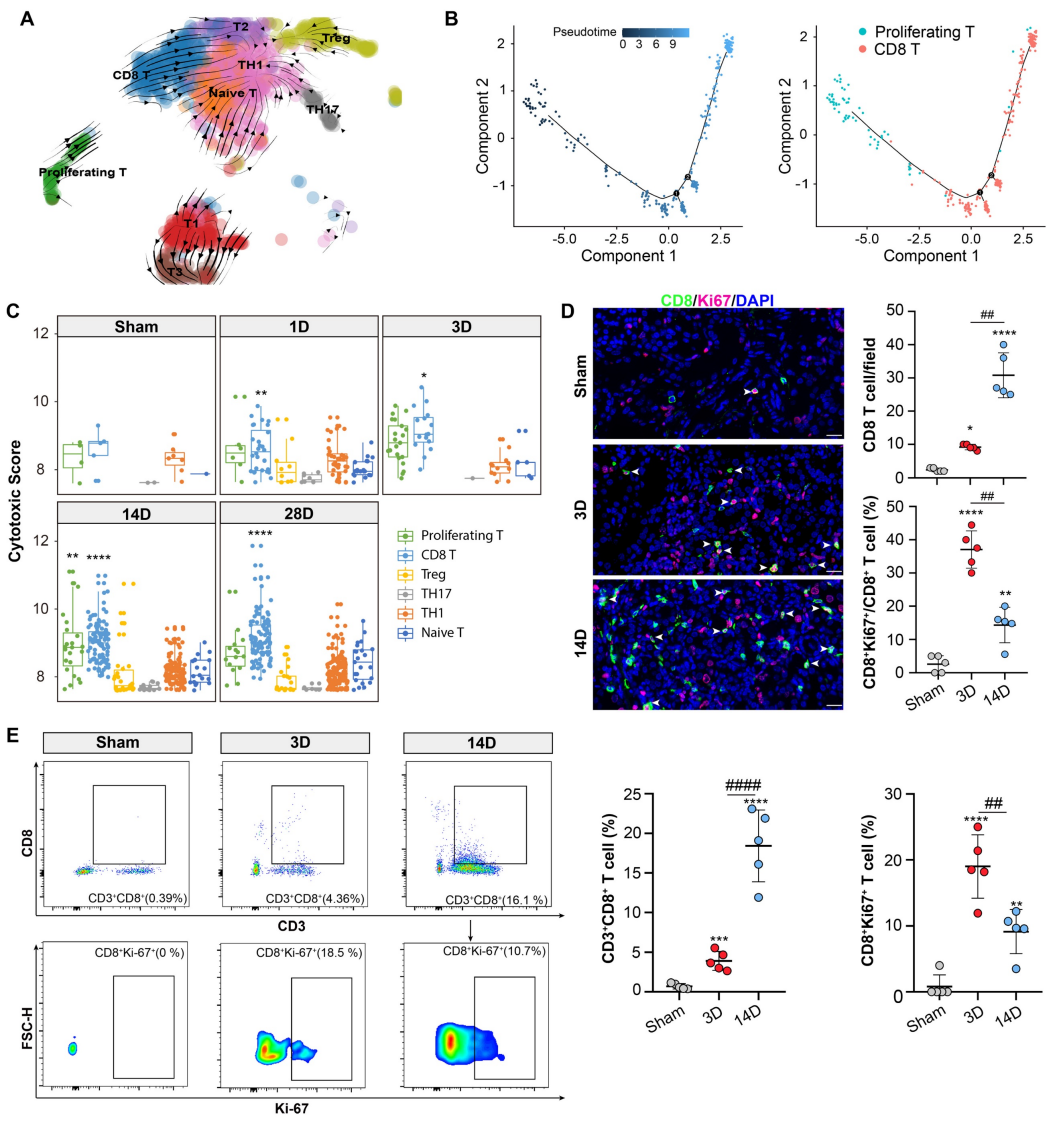
** Proliferative T cluster differentiates into CD8 T cluster with cytotoxic function.** (A) RNA velocity analysis overlaid on the UMAP plot illustrating the major developmental paths of T cell subtypes after AKI. (B) Pseudo-time trajectory analysis overlaid on the UMAP plot depicting the developmental paths of proliferating and CD8 T cells after AKI. (C) Cytotoxic scores of T cell subtypes at each time point of the uIRI model. (D) Representative immunofluorescence images of CD8^+^ and CD8^+^Ki-67^+^ cells in 3D or 14D after AKI(n=5). (E) Flow Cytometry results of CD8 and CD8^+^Ki-67^+^ T cells proportion in 3D or 14D after AKI(n=5). Scale bars, 20 μm. Data are presented as means ± SD. *P < 0.05, **P < 0.01, *** *P* < 0.001, **** *P* < 0.0001, compared to the sham group or Naive-T cluster; ^##^ *P* < 0.01, ^####^ *P* < 0.0001, compared to uIRI 3D model.

RNA velocity and pseudotime analysis were employed to explore the trajectories of the CD8 and proliferating T cell cluster. Both analyses revealed the transcriptional differentiation from the proliferating T cluster to the CD8 T cluster (Figures 3A and 3B). Accordingly, the proliferating T cluster displayed high expression levels of Mki67 and Top2a, while the CD8 T cells showed elevated expression levels of Ccl5, Gzmk, and Nkg7 (Figures S2A and S2B). Recent study has revealed that Gzmk-positive CD8 T cells have the potential to drive inflammation, and form a core population in multiple inflamed human tissues 21. Notably, Ccl5 and Nkg7 are known hallmarks of CD8 effector cells, indicating the activation of CD8 T cluster during the chronic progression of AKI 22. CD8 effector cells are also referred to as CD8 cytotoxic T lymphocytes (CTL). Correspondingly, the CD8 T and proliferating T cluster exhibited the highest cytotoxic scores compared to the other clusters (Figure 3C).

Next, immunofluorescence staining confirmed the presence of a CD8 positive proliferating T cluster (CD8^+^ Ki-67^+^ T cluster) (Figure 3D). Importantly, a remarkable accumulation of CD8^+^ Ki-67^+^ T cluster was observed on day 3 after uIRI which might be the critical phase for the chronic transition, CD8 T cells were more abundant thereafter at 14D after AKI (Figure 3D). Additionally, flow cytometry results also confirmed the above proportion changes of CD8 T and CD8^+^ Ki-67^+^ T cluster in 3D and 14D after AKI (Figure 3E; Figure S3A). Therefore, those data collectively demonstrated that the CD8 T cells originated from the proliferating T cluster at the early stage after AKI which may be crucial for the chronic transition of AKI through its remarkable cytotoxic function on target cells.

### Proliferating/CD8 T cluster is recruited by macrophages through Cxcl16-Cxcr6 signaling

**Figure 4:**
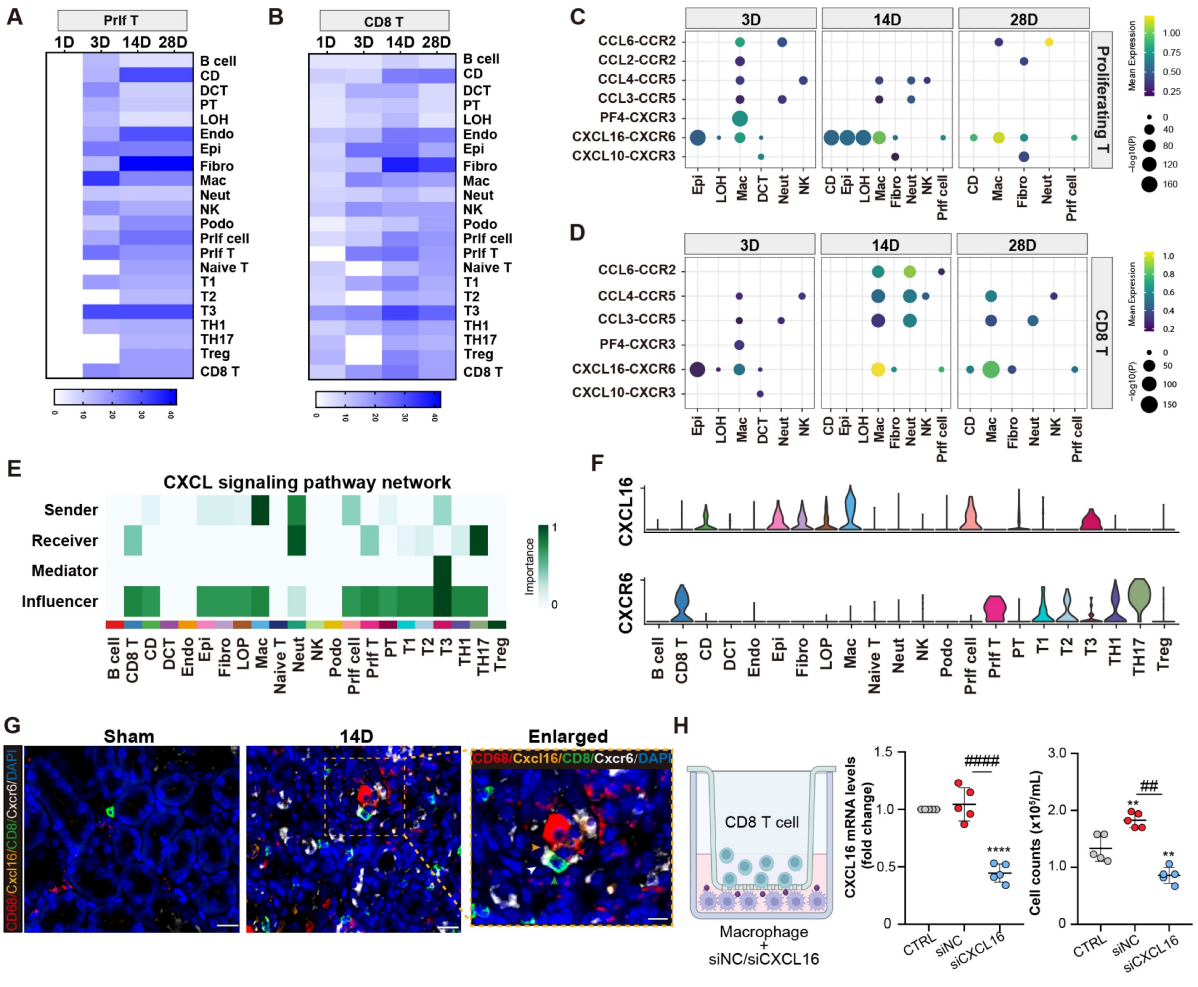
** Proliferating/CD8 T cluster is recruited by macrophages through Cxcl16-Cxcr6 signaling.** (A-B) The interaction numbers between proliferating/CD8 T subtypes and other cells at each time point. (C-D) Enriched numbers of cytokine and chemokine signaling pathway interactions between other cells and proliferating/CD8 T subtypes. (E-F) Enriched CXCL signaling pathway networks and the significant ligand-receptor pair CXCL16-CXCR6 between macrophages and CD8 T cluster are depicted (CD: collecting-duct; DCT: distal convoluted tubule; PT, proximal tubule; LOH: loop of Henle; Endo: endothelial cell; Epi: epithelial cell; Fibro: fibroblast; Mac: macrophage; Neut: Neutrophil; NK: natural killer Cell; Podo: podocyte; prlf cell: proliferation cell.). (G)Representative immunofluorescence images of the spatial distribution of CXCL16, CD68 positive macrophages alongside CXCR6 positive CD8 T cells. (H) A co-culture system was applied to evaluate the migration capacities of the CD8 T cell cluster when affected by macrophages transfected with CXCL16 siRNA or NC siRNA. The number of migrated CD8 T cells in the lower chamber was quantified. Scale bars, 20μm (Enlarged Scale bars, 10μm.). Data are presented as means ± SD.**P<0.01, **** *P* < 0.0001, compared to the control group; ^##^ *P*<0.01, ^####^ *P*<0.0001, compared to macrophages transfected with NC siRNA group.

An analysis of receptor-ligand interaction was conducted to elucidate the factors contributing to the recruitment of the CD8 T cells following AKI. It was revealed that fibroblasts, collecting-duct, endothelial cells, and macrophages exhibited significant interactions with the proliferating/CD8 T cluster. Notably, macrophages displayed the strongest interaction with the proliferating T cluster at day 3 post-AKI (Figures 4A and 4B). A number of cytokine and chemokine pathways between macrophages and the proliferating/CD8 T clusters were observed (Figures 4C and 4D). Moreover, the Cxcl16-Cxcr6 signaling pathway network was enriched in macrophages and the proliferating/CD8 T clusters after AKI. Importantly, macrophages served as the primary source of the CXCL signaling pathway, whereas the proliferating/CD8 T cluster served as the important recipients of this pathway (Figure 4E). Further ligand-receptor analysis also confirmed the importance of the Cxcl16-Cxcr6 signaling pathway network between macrophages and the proliferating/CD8 T clusters (Figure 4F).

Next, multiplex fluorescence staining confirmed a close proximity and interaction between Cxcl16, CD68 positive macrophage, and CD8, Cxcr6 positive T cell (Figure 4G). To further investigate the role of macrophages in recruiting the CD8 T cells *in vitro*, a transwell co-culture system was employed, where the CD8 T cells were placed in the upper chamber, and macrophages treated with either Cxcl16 siRNA or scramble control were placed in the lower chamber. Impressively, Cxcl16 siRNA-treated macrophages significantly reduced the migration of CD8 T cells compared to the control group (Figure 4H). These results demonstrate the critical role of macrophages in recruiting the proliferating/CD8 T clusters through cytokines and chemokines, especially Cxcl16-Cxcr6 interaction.

### CD8 T cells induce endothelial cell apoptosis through Fasl-Fas signaling

**Figure 5:**
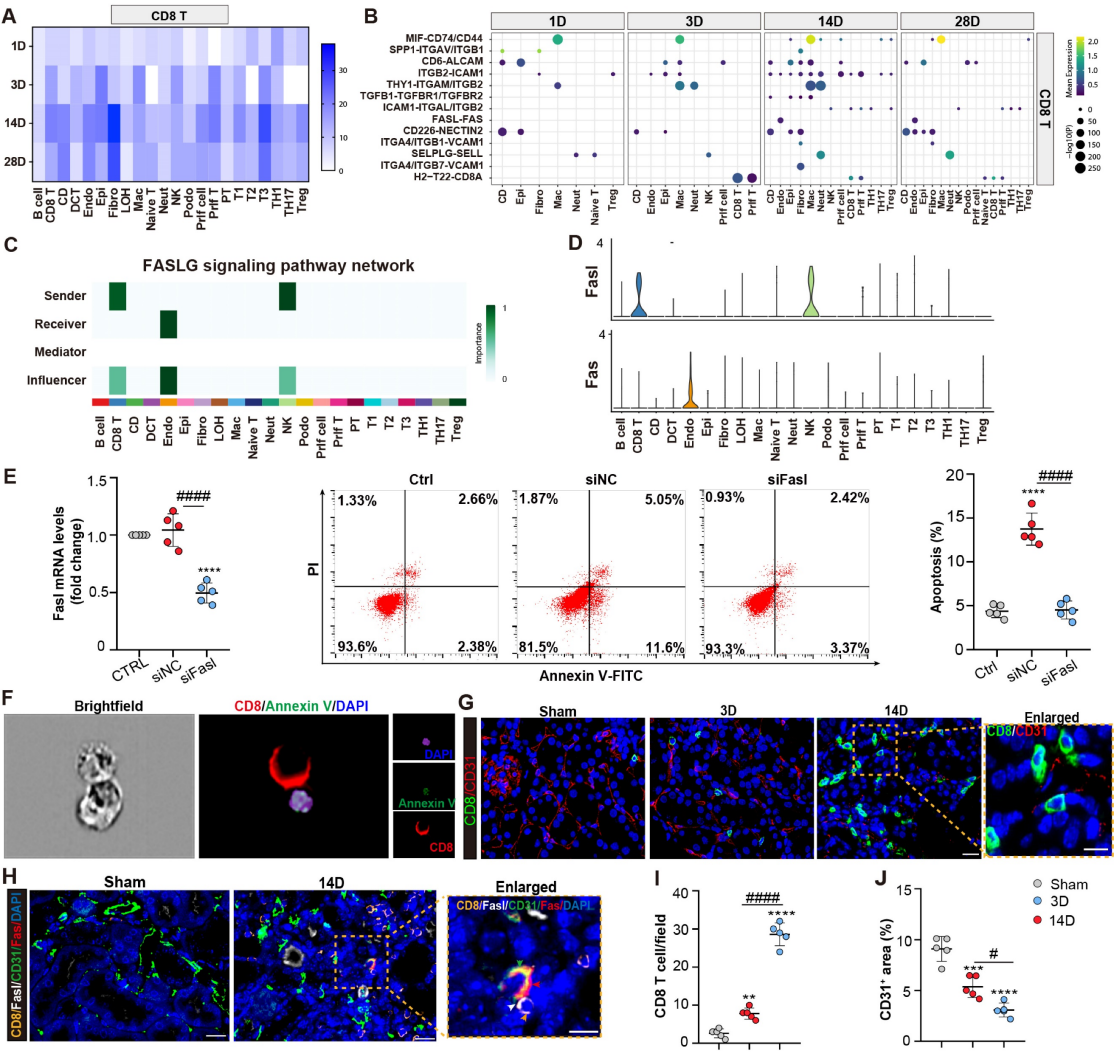
** CD8 T cells induce endothelial cell apoptosis through Fasl-Fas signaling.** (A) The interaction numbers between the CD8 T cells and other cells at each time point. (B) The pathway network between the CD8 T cells and other cells. (C-D) Enriched FASLG signaling pathway networks and significant ligand-receptor pair Fasl-Fas in CD8 T and endothelial cluster. (E) Flow cytometry results of the apoptosis of endothelial cells, when co-cultured with CD8 T cells, transfected with Fasl siRNA or NC siRNA. (F) Representative images showing CD8 positive T cells attach to Annexin V and DAPI positive endothelial cells using ImageStream®X Mk II. (G) Representative immunofluorescence images of the number and location of CD8 T cells and CD31^+^ area in 3D and 14D uIRI model. (H) Representative immunofluorescence images of the spatial distribution of Fasl positive CD8 T cells, alongside Fas, CD31 positive endothelial cells. (I, J) Quantifying the number of CD8 T cells and CD31^+^ area in 3D and 14D uIRI model (n=5). Scale bars, 20μm (Enlarged Scale bars, 10μm.). Data are presented as means ± SD. **P<0.01, ***P<0.001, ****P<0.0001, compared to control or sham group; ^#^ *P*<0.05, ^####^ *P*<0.0001, compared to CD8 transfected with NC siRNA group or 3D uIRI model.

We next investigated the role of the CD8 T cells in the chronic progression of AKI. Receptor-ligand interaction analysis showed that CD8 T cells increased their interactions with different fragments of tubules, endothelial, and fibroblast cells with the chronic progression of AKI (Figures 5A and 5B). Importantly, the Fasl -Fas interaction pathway was identified as specific to CD8 T cells and endothelial cells at 14 and 28D in the chronic period. Pathway contribution visualization and gene violin expression analyses further confirmed the significant role of the FASL pathway, with endothelial cells being the exclusive recipients, indicating the pro-apoptosis effects as a consequence (Figures 5C and 5D).

Next, primary endothelial cells are co-cultured with CD8 T cells transfected with either Fasl siRNA or scramble control. Apoptosis of endothelial cells was induced when co-culture with the CD8 cells, which was largely reversed by Fasl siRNA treatment in CD8 T cells (Figure 5E; Figures S4A and 4B). Additionally, an ImageStream X Amnis System was utilized to visualize the pro-apoptotic function of CD8 T cells on endothelial cells (Figure 5F; Figure S3B). Interestingly, compared to D3 after AKI, increasing CD8 T cells were infiltrated which localized close to the endothelial cells, accompanied with decreased density of CD31^+^ capillaries at 14D in uIRI model (Figures 5G, 5I, and 5J). Impressively, fluorescence staining confirmed the accumulation of Fasl-positive CD8 T cells around Fas-positive endothelial cells in kidney tissue at 14D after uIRI surgery (Figure 5H). Taken together, these findings demonstrated that CD8 T cells induced apoptosis of endothelial cells through Fasl-Fas signaling during the chronic transition of AKI.

### CD8 T cells cause rarefaction of peritubular capillaries (PTCs) and renal fibrosis

**Figure 6:**
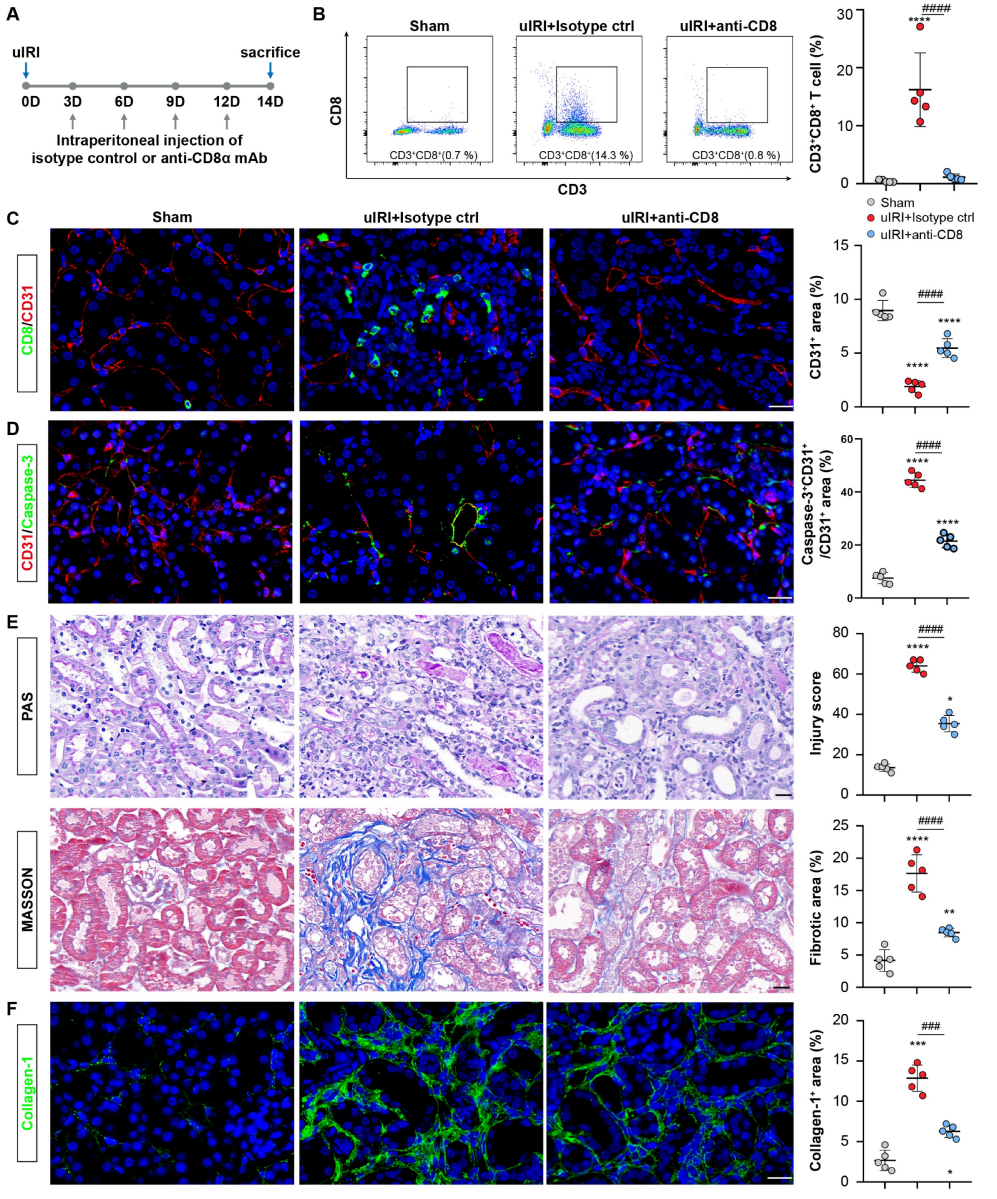
**CD8 T cells cause rarefaction of peritubular capillaries (PTCs) and renal fibrosis.** (A)The flow chart of the experimental. (B) Flow cytometry results of the proportion of CD8 T cells in sham, uIRI 14D model treated with CD8α mAb or Isotype control group (n = 5). (C) Representative immunofluorescence images of the number of CD8 T cells and CD31^+^ area in sham, uIRI 14D model treated with anti-CD8α mAb or Isotype control group. (D) Representative immunofluorescence images of the apoptosis of CD31^+^ cells in sham, uIRI 14D model treated with CD8α mAb or Isotype control group. (E) Representative histopathology images of PAS and Masson staining in sham kidneys, uIRI 14D model treated with CD8α mAb or the Isotype control group. (F) Representative immunofluorescence images of the positive area of collagen-1 staining in sham kidneys, uIRI 14D model treated with CD8α mAb or Isotype control group. Scale bars, 20μm. Data are presented as means ± SD. * *P*<0.05, **P<0.01, *** *P* <0.001, **** *P* <0.0001 compared to sham group;^ ###^ *P* <0.001, ^####^* P* <0.0001 compared to uIRI model treated with Isotype Control.

**Figure 7:**
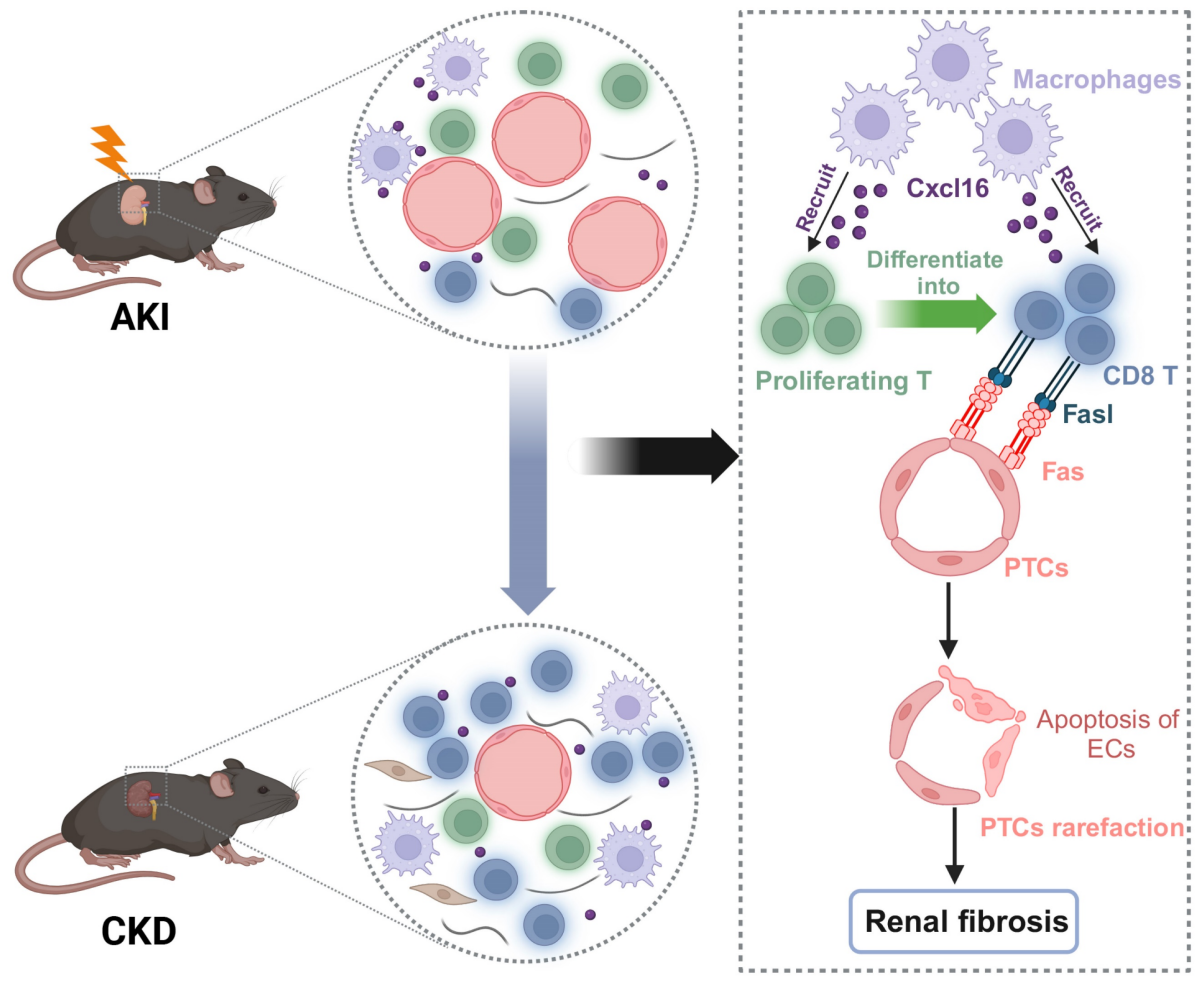
** Working models propose CD8 T cells mediate the apoptosis of endothelial cells, which leads to the rarefaction of PTCs and renal fibrosis after AKI.** We demonstrated that CD8 T cells were recruited by macrophages through Cxcl16-Cxcr6 signaling. CD8 T cluster originated from the proliferating T cluster and induced apoptosis of endothelial cells through Fasl-Fas signaling after AKI, eventually leading to the rarefaction of PTCs and renal fibrosis.

The rarefaction of PTCs is a critical pathological characteristic of renal fibrosis 23. To investigate the role of CD8 T cells in capillaries rarefaction *in vivo*, CD8 T cells were eliminated by administering an anti-mouse CD8a antibody every 3D in uIRI model, while an isotype control was administered as the control (Figure 6A). Following the injection of anti-mouse CD8α antibody, CD8 T cells were almost eliminated (Figure 6B and 6C; Figure S3C). Impressively, PTCs rarefaction was remarkable in the location where CD8 T cells infiltrated in uIRI mice, while elimination ameliorated the apoptosis of endothelial cells and reversed the loss of CD31^+^ area significantly at 14D after uIRI (Figures 6C and 6D). Meanwhile, the renal injury was alleviated as shown by reduced tubular casts, tubular necrosis, and a significant decrease of the fibrotic area following treatment with anti-mouse CD8α antibody (Figures 6E and 6F). Correspondingly, WB and qPCR results confirmed renal collagen-1and α-SMA were decreased after treatment with anti-mouse CD8α antibody (Figures S5A-5E). Therefore, these results provide evidence that CD8 T cells induced the apoptosis of endothelial cells, resulting in the rarefaction of PTCs and ultimately promoting the chronic transition of AKI (Figure 7).

## Discussion

T cells consist of multiple subtypes, each with diverse role in kidney diseases 24. However, the dynamic landscape and the ontogeny of T cell subtypes have not been thoroughly investigated during the transition from AKI to CKD. In the present study, we illustrated the dynamic profile of T cells and identified that CD8 T cells present as the main immune cell population derived from the proliferating cluster, which leads to the apoptosis of endothelial cells and eventually PTCs rarefaction.

Firstly, we delineated the landscape of T cells in the process of AKI to CKD transition by using scRNA-seq analysis. We found that the CD8 T cells present as the main population which increased remarkably at day 1 and displayed a second peak at 14D after AKI. Importantly, it was demonstrated that CD8 T cells originated from the proliferating T cells with the highest proliferation response at day 3 after AKI, highlighting the critical role of T cells with proliferative capacity. Local proliferation of activated T cells was also observed in SARS-CoV-2 pneumonia, which drives persistent alveolar inflammation 25. Similarly, proliferating T cells were identified in the injured kidneys at a late stage after IRI, with both neutrophils and T-cell populations markedly increased at 14D through integrated scRNA-seq analysis 19. Herein, we further identified the proliferative response of those T cell clusters at day 3 after IRI and emphasized their contribution to the late accumulation of T cells. Unlike the traditional understanding that renal T cells exclusively originate from the circulating system, recent studies have identified Tissue-resident-memory T (TRM) cells, a non-circulating subset of lymphocytes, primarily implicated in renal autoimmune diseases 26,27. It would be of significance to further clarify the contribution of TRM in PTCs rarefaction after AKI. In this study, our study highlighted the dynamic landscape of T cells and identified that proliferating T cells present at day 3 after AKI as the early cell responders contribute to the second wave of inflammation characterized by T cell accumulation in the chronic period.

The kidney interstitium is inhabited by innate immune cells and T cells under normal circumstances, and cell-cell interaction may contribute to the establishment of a proinflammatory microenvironment after injury. We identified that macrophages played a pivotal role in recruiting CD8 T cells through chemokines, especially the Cxcl16-Cxcr6 pathway. Interestingly, the strong interaction between Cxcl16^+^ macrophages and Cxcr6^+^ CD8 T cells was also observed in renal allograft rejection 28. Notably, our results demonstrated that inhibiting Cxcl16 on macrophages could impede the migration of CD8 T cells remarkably.

Indeed, the inhibition of Cxcl16 could reduce the production of pro-inflammatory molecules following AKI, indicating the essential role of Cxcl16 in the formation of pro-inflammatory microenvironment 29. Currently, there are no antibodies available for clinical use specifically targeting CXCL16. A prior study has demonstrated that recombinant tumor necrosis factor receptor: Fc fusion protein (rhTNFR: Fc) was able to mitigate inflammation in patients with ankylosing spondylitis by inhibiting the Cxcl16/Cxcr6 pathway 30. However, the potential efficacy of rhTNFR: Fc in downregulating Cxcl16 expression in macrophages during the chronic progression of AKI warrants further exploration. The findings from this study underscore the crucial role of macrophages in the recruitment of CD8 T cells, macrophage derived Cxcl16 may represent a promising target in impeding the secondary peak inflammation mediated by T cells in the chronic stage of AKI.

PTCs rarefaction is one of the major hallmarks of CKD and an important pathway for AKI transition to CKD 31,32. However, the mechanisms driving the capillary loss remain largely unclear. Previously, the diminished vascular endothelial cell survival factors, malfunction of endothelial cells and endothelial progenitor cells, and pericyte detachment were considered as the events that lead to PTCs rarefaction 33. A local alteration in the balance between angiogenic and angiostatic factors is usually regarded as the major mechanism and therapeutic target for PTCs rarefaction 34. However, the correlation of persistent inflammation mediated by multiple immune cells in PTCs loss is neglected. Our study identified a novel mechanism by which CD8 T cells induced the apoptosis of endothelial cells via the Fasl-Fas pathway, subsequently leading to the PTCs rarefaction. Indeed, T cells have been observed to accumulate in the vasculature by adhering to vascular endothelial cells in the early phase of reperfusion after stroke 35. In renal allograft recipients, PTCs dilation is strongly correlated with intracapillary inflammation, suggesting the role of inflammation in the pathogenesis of PTCs dilation 36. Therefore, CD8 T cell mediated inflammation is identified as the previously unrecognized factor that is involved in PTCs loss after AKI. The interaction of CD8 T cells and endothelial cells leads to PTCs rarefaction and renal fibrosis, which may provide a novel therapeutic target for preventing the chronic transition of AKI.

Although treatment with angiogenic growth factors and endothelial progenitor cells, strengthening of the signaling that was essential for the recruitment of pericytes to vasculature are proposed as potential strategies for protection against capillary rarefaction, while practical therapeutic tools are still lacking. In this study, we identified that depletion of CD8 T cells using anti-CD8α mAb could significantly attenuate PTCs rarefaction and consequent renal fibrosis. Recent studies have identified that renal function and inflammation are greatly ameliorated after anti-CD8 mAb treatment in the Adriamycin mouse model 37,38. Allograft survival of cynomolgus monkeys was largely improved after depleting the CD8 memory T cells 39. Given these findings, depleting CD8 T cells may be a new effective intervention approach to halt the progression of CKD. The Food and Drug Administration (FDA) has approved TZIELD (teplizumab-mzwv), a monoclonal antibody targeting CD3, for the treatment of type 1 diabetes (T1D) 40. More importantly, recent studies have revealed that TZIELD could induce the exhaustion of CD8 T cells 41,42. The potential of TZIELD in suppressing the deleterious effects of CD8 T cells on PTCs deserves further investigation. Collectively, our findings highlight the potential of CD8 T cells as a novel therapeutic target for preventing the chronic transition after AKI via protection of PTCs integrity.

In conclusion, the present study demonstrated that CD8 T cells originating from the proliferating cluster contributed to the second peak of inflammation in the chronic stage of the progression after AKI. Our finding provides a novel mechanism of PTCs rarefaction induced by CD8 T cells during the chronic progression after AKI, which may provide new intervention strategy for preventing AKI progressing to CKD.

## Materials and Methods

### Animals

C57BL/6J mice, aged six to eight weeks, were obtained from the Animal Experimental of Vital River. All animals were conducted following the recommendations specified in the Guide for the Care and Use of Laboratory Animals of the National Institutes of Health. The protocol received approval from the Committee on the Ethics of Animal Experiments of Southeast University. For the unilateral ischemia-reperfusion injury (uIRI) model, mice were anesthetized with isoflurane and subjected to ischemia by clamping the unilateral renal pedicles using non-traumatic microaneurysm clamps for 35 minutes, followed by reperfusion to restore renal blood flow by removing the clamps as reported before 43,44. The sham group underwent a sham surgery, and their kidneys were harvested. The remaining kidneys were harvested on 1, 3, 14, and 28D after the uIRI procedure. In the CD8 depletion experiment, mice were injected intraperitoneally with 250 μg/mouse neutralizing mAbs directed against CD8 (clone 2.43; BioXcell) every 3D 45. The control group was injected intraperitoneally with the corresponding isotype (BioXcell). Finally, mice were sacrificed after 14D to proceed with further analysis.

### scRNA-Seq by 10× genomics

The collected cells were acquired from three mice's kidneys (n=3 uIRI samples each day and n=3 control samples) and then pooled together to create a single sample for each group, which was used for subsequent analysis. For single-cell RNA sequencing (scRNA-seq), tissue was subjected to single-cell isolation as previously 46. Cell number and viability were determined by Countstar (Alit Biotech, Rigel S2). This approach produced a single-cell suspension with more than 90% viability. The prepared cell suspension was added into the Chromium single-cell controller (10x Genomics, GCG-SR-1) to generate single-cell GEMS (Gel Bead-In-EMulsions) according to the manufacturer's instruction by utilizing the Single Cell G Chip Kit (10x Genomics, 1000120). Reverse transcription was done on an S1000TM Touch Thermal Cycler (Bio-Rad) following the manufacturer's procedure. The cDNA was generated and tested for quality using an Agilent 4200. Then single cell 3' Library and gel bead kit V3.1 were utilized to generate single-cell RNA-seq libraries. Eventually, the libraries were sequenced on an Illumina Novaseq 6000 sequencer with more than 100,000 reads per cell and pair-end 150 bp (sPtrEa1t5e0gy) (completed by CapitalBio Technology).

### scRNA-seq data processing, quality control, and analysis

The sequencing raw data was processed into files following the standard Chromium's Cell Ranger pipeline as reported before 47. Filtering and unique molecular identifier (UMI) counting were performed using the Cell Ranger single-cell software suite, resulting in a filtered gene-cell matrix. The gene-cell data matrices were imported into the R package (v4.2) for identification using the Seurat package. Subsequently, all uIRI and sham samples were pooled, and poor-quality cells with 200 expressed genes and mitochondrial gene percentages greater than 25 were excluded. In addition, cells recognized as doublets or multiplets were removed using the DoubletFinder function, of which several marker genes were highly expressed in a single cell. Then, 2000 highly variable genes were performed for principal component analysis (PCA). Uniform manifold approximation and projection (UMAP) were employed for visualization. The Harmony package was utilized to integrate gene-cell matrices from different specimens. Cell types were determined by comparing cluster-specific markers with known cell subtype signature genes in the cell marker database and previous research publications.

### Pseudo-temporal and RNA velocity analysis

RNA velocity analysis was performed using the velocity and scvelo python packages to validate the developmental trajectory of T cells from AKI to CKD. The files were converted to loom format. RNA velocity was then calculated utilizing the steady-state model with the stochastic option 48. RNA velocity was embedded into the UMAP plots using the velocity graph. Monocle2 analysis was performed to confirm the relationship between T clusters. The cells were assigned a pseudo-time trajectory based on the union of highly variable genes in a set of principal components used for time course analysis of T clusters. The branch-dependent genes were identified using the branched expression analysis modeling (BEAM) function in Monocle2, and they were classified into two clusters based on pseudo time.

### Cell-cell interaction network analysis

A draft network was utilized to study ligand-receptor interactions across different cell types. Cell-cell communication calculation and analysis were performed using the R package cell-chat with default parameters 49. In brief, ligand-receptor pairs were selected between T clusters and other cells. The interaction specificity and intensity were defined based on the level of ligand color in a cell type and the level of cognate receptor color in another cell type simultaneously.

### Calculation of cytotoxic scores

The cytotoxic gene set involved the following genes which were also reported in the previous study: PRF1, IFNG, NKG7, GZMB, GZMA, KLRK1, KLRB1, KLRD1, CTSW, CST7, and GZMK 50. The cytotoxic scores were calculated through the Add Module Score function of Seurat packages. The compare mean cytotoxic function was utilized for calculating p-value of two clusters by Wilcoxon rank test.

### Kidney histology and Immunofluorescence

Kidneys were fixed with 4% paraformaldehyde, embedded in paraffin, and sectioned into 4 μm thick slices for Periodic Acid-Schiff (PAS), Masson's trichrome, immunohistochemistry and immunefluorescence staining. The Histology of the tubular in each grid was semi-quantitatively calculated as previously mentioned 51. Five random sections of each mouse were selected to assess the degree of renal fibrosis, and then the fibrotic area percentage was calculated by defining the area of blue staining. For multiple Immunofluorescence, after deparaffinization and rehydration, the fixed kidney sections were analyzed using an Alpha TSA Multiplex IHC Kit (Alpha X Biotech CO.) according to the manufacturer's instructions. The primary antibodies against CD8 (GB114196, Service bio), CD68 (GB113109, Service bio), Cxcl16 (bs-1441R, Bioss), Cxcr6 (PA579117, Thermo Fisher), CD31 (GB113151, Service bio), Fasl (GB113151, Service bio), Fas (RT-1124, Hua bio) was utilized, followed by incubation with corresponding secondary antibodies. Image Pro Plus image analysis system was then utilized to quantify the number of CD8 T cells and the percentage of CD31^+^ area.

### Cell culture and reagents

Primary mouse glomerular endothelial cells were purchased from the Shanghai Institute of Biochemistry and Cell Biology (SIBCB), Chinese Academy of Sciences. Raw264.7 macrophages were from American Type Culture Collection (ATCC). The isolation of mouse CD8 T cells was described previously 52. Briefly, CD8 T cells were isolated from the spleen of C57BL/6 mice by CD8 T Cell Isolation Kit (Miltenyi Biotec) and activated with a final concentration of 2 mg/ml anti-CD3/CD28 monoclonal antibody for subsequent use. Raw264.7 macrophages and CD8 T cells were cultured in RPMI 1640 media containing 10% fetal bovine serum (Science Cell) and 100 mg/ml penicillin-streptomycin(P/S). Primary mouse glomerular endothelial cells were cultured in endothelial cell medium (ECM) (Science Cell) containing 10% fetal bovine serum and 100 mg/ml P/S. All cell lines were cultured at 37 °C in a 5% CO2 incubator.

### Flow cytometry

Mice were euthanized, and the single-cell suspension of the kidney was prepared by using the multi-tissue dissociation kit 2 (Miltenyi Biotec, Cat. No. 130-110-203) according to manufacturers' instruction and an octo MACS tissue dissociator (Miltenyi Biotec). The cell suspensions were incubated with Fc block (BD Biosciences, Cat. No.553141) for 15 minutes on ice. Cells were treated with the Live/DEAD-Fixable Viability Stain 780 (BD Biosciences, Cat. No. 565388) for 15 minutes on ice and washed twice. Then cells were incubated with the following antibodies: CD45-Brilliant Violet 510 (BD Biosciences, Cat. No. 563891), CD3-Brilliant Violet 421 (BD Biosciences, Cat. No.562600), CD8-PerCP-Cy™5.5 (BD Biosciences, Cat. No. 551162) for 30 minutes on ice. Cells were then washed two times and fixed (BD Cytofix/Cytoperm TM, BD Biosciences, Cat. No.554714) and incubated with Ki-67-Alexa Fluor™ 700 (eBioscience™, Cat. No.56-5698-82). Additionally, cell apoptosis was detected using the Annexin V-FITC apoptosis detection kit (KGA1102, Keygen). Briefly, cells were collected and cell suspensions were prepared. Then, the cells were washed twice with PBS. Then cells were incubated with fluorescein isothiocyanate (FITC) - Annexin V and propidium iodide (PI) antibody for 15 mins. Flow cytometry was performed on a FACSymphony A5 SORP (BD Biosciences), and data were analyzed by FlowJo 10.8.

### Image stream analysis

CD8 T cells were isolated and stimulated as previously described, then incubated with endothelial cells for 12h. The cell suspensions were collected, washed twice, and then incubated with Fc block (BD Biosciences, Cat. No.553141) for 15 minutes. Then cells were treated with the following antibodies: CD8-APC (BD Biosciences, Cat. No. 553035) and Annexin V-FITC (eBioscience™, Cat. No. BMS147FI) for 30 minutes, and then were washed and resuspended in 200μL of PBS. Dapi was added to the cell suspensions before examination and then tested using the Amnis ImageStream MK II. Results were analyzed by IDEAS analysis software (Amnis Corporation).

### RNA preparation and Quantitative Real-Time Polymerase Chain Reaction (qRT-PCR)

According to the manufacturer's instructions, the procedure was reported previously 53. The primers were as follows: Mouse (Mus) collagen-1 Forward primer: 5′GGACGCCATCAAGGTCTACT3′; Reverse: 5′GAATCCATCGGTCATGCTCT3′; Mus Acta2 Forward primer: 5′CCCAGACATCAGGGAGTAATGG3′; Reverse: 5′TCTATCGGATACTTCAGCGTCA3′; Mus Fasl Forward primer: 5′ACCACCTCCATCACCACTACC3′; reverse 5′CATTCCAA CCAGAGCCACC3′; Mus GAPDH Forward primer: 5′GCATGGCCTTCCGTGTTC3′; Reverse: 5′GATGTCATCATACTTGGCAGGTTT3′.

### Western blotting

Total protein extraction and western blotting of renal tissues were performed as previously described 53. The antibodies were utilized as follows: anti-Collagen-1 (bs-7158R, Bioss), anti-α-SMA (ab7817, Abcam), anti-Gapdh (Ab2000, Abways). The secondary antibodies were goat anti-mouse HRP (FDM007, FDbio science) and goat anti-Rabbit HRP (FDR007, FDbio science). Target proteins were normalized to GAPDH levels. Finally, an ECL chromogenic substrate was applied to detect the fluorescent signals (BIO-RAD, USA).

### Statistics

Statistical analyses were conducted using a t-test or one-way analysis of variance (ANOVA) in Prism 8.0 GraphPad Software. Data are presented as means ± SD. Statistical significance was defined as *P < 0.05.

## Supplementary Material

Supplementary figures and table.
