# Supplementary material for: CD8 T cells induce the peritubular capillary rarefaction during AKI to CKD transition
Source: Int J Biol Sci. 2024 May 19;20(8):2980–93. doi: 10.7150/ijbs.96812 (PMC11186369; doi:10.7150/ijbs.96812)
Supplement: Supplementary file 1 — Supplementary figures and table. [file ijbsv20p2980s1.pdf]

SUPPLEMENTARY MATERIALS:

Supplementary Table 1. **Gene information in the enriched pathway of CD8 T cells after GO analysis.**

Term	Count	PValue	Genes
GO:0002376~Immune system process	18	3.24E-10	SLFN2, CD160, H2-Q7, SH2D1A, ARID5A, THEMIS, CD8B1, CD3G, SERPINA3G, CD3E, CD3D, PSMB8, PSMB9, JAML, CD8A, CD7, PDCD1, SKAP1
GO:0002250~Adaptive immune response	13	3.86E-09	SLFN2, CD160, SH2D1A, THEMIS, CD8B1, CD3G, SERPINA3G, CD3E, CD3D, CD8A, CD7, PDCD1, SKAP1
GO:0006915~Apoptotic process	9	0.009337	FASL, IFI27, SH3KBP1, BCL2, CD27, GZMB, PDCD1, LSP1, SERPINA3G
GO:0007155~Cell adhesion	8	0.019671	SELPLG, ITGA4, JAML, LAMB3, ITGA1, CD226, ITGAE, THY1
GO:0006955~Immune response	7	0.030113	FASL, CCL5, H2-Q7, H2-M3, CXCR6, CST7, CCR5
GO:0006508~Proteolysis	7	0.050915	GZMK, GZMB, CTSW, KLK8, PSMB8, CTSC, PSMB9
GO:0051603~Proteolysis involved in cellular protein catabolic process	6	1.89E-05	CTLA2A, GZMB, CTSW, PSMB8, CTSC, PSMB9
GO:0034097~Response to cytokine	6	3.14E-05	SP100, CCL5, BCL2, ACP5, SERPINA3G, CORO1A
GO:0032729~Positive regulation of interferon-gamma production	6	4.43E-05	CD160, H2-M3, ARID5A, CD27, CD226, CD3E
GO:0006935~Chemotaxis	6	3.64E-04	CMTM7, CCL5, RAC2, CXCR6, LSP1, CCR5

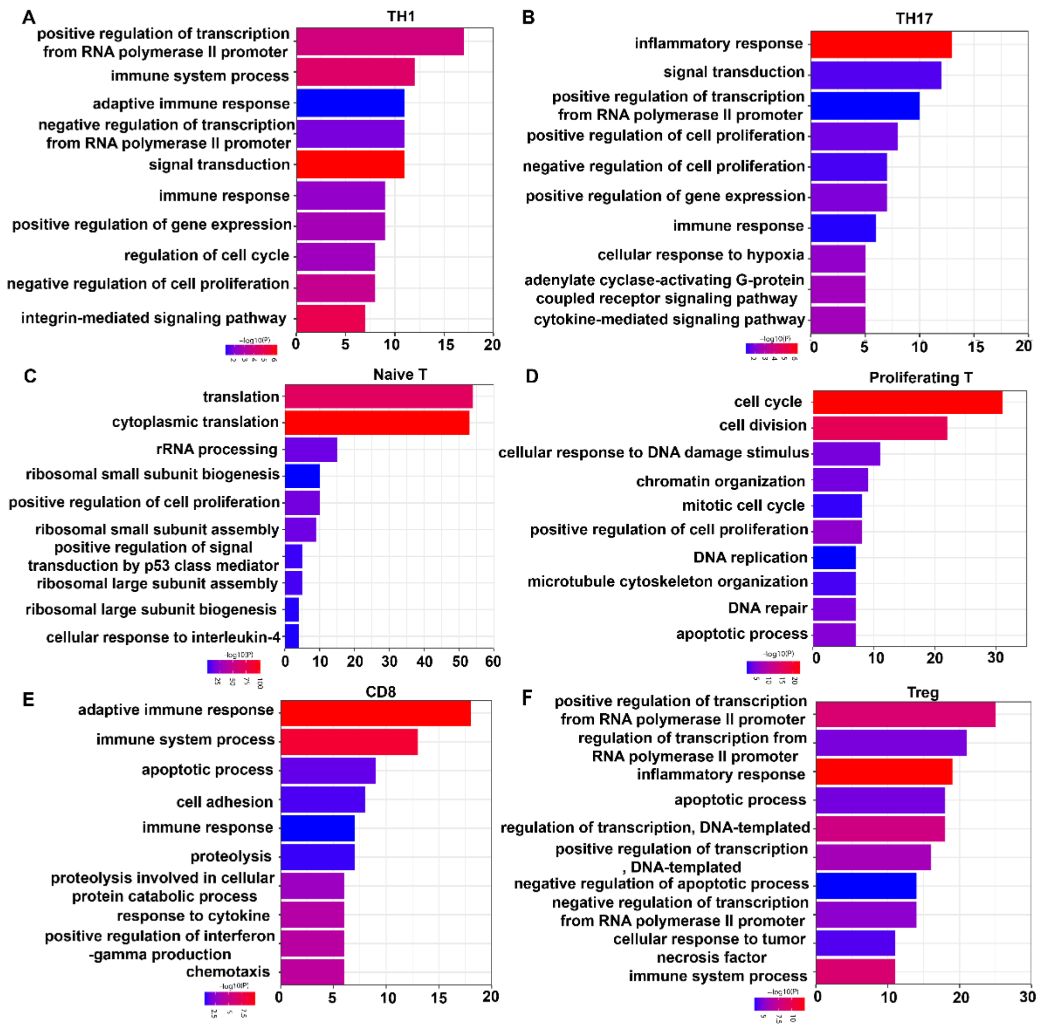

Supplementary Figure 1. **The disparate function of T cell clusters during AKI to CKD.**

(a-f) GO pathway analysis of the top 10 upregulated pathways in TH1, TH17, naive, Proliferating T, CD8, and Treg cluster, ranked by *counts*.

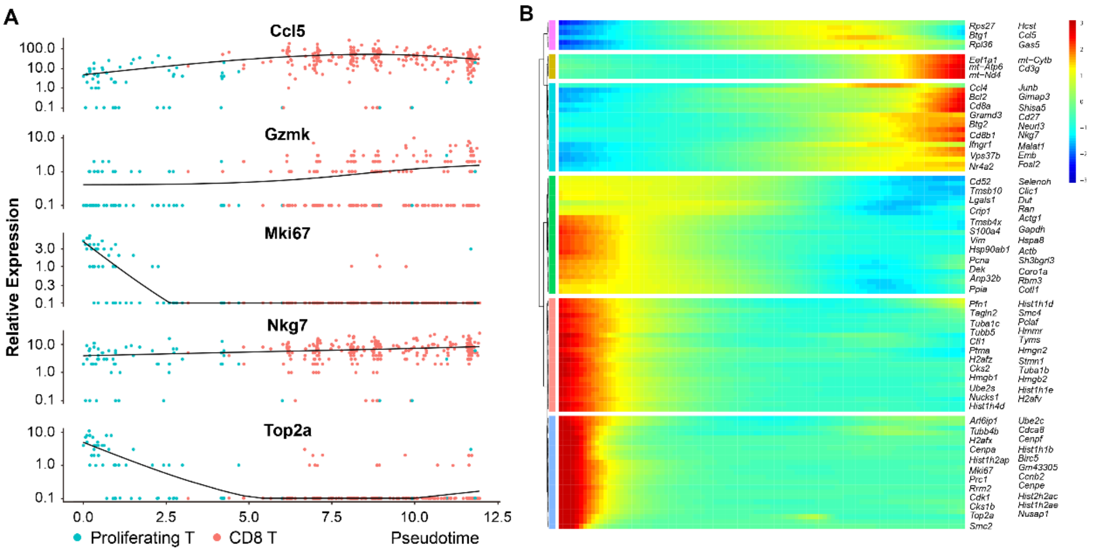

Supplementary Figure 2. **The dynamic changes of important genes and the pseudo time during the transition of proliferating T to CD8 T cells.**

(a) Expression dynamics of unique representative genes Ccl5, Gzmk, Mki67, Nkg7, and Top2a analyzed using Monocle 2 after AKI. (b) Heatmap showing important genes involved in cell-state transitions after AKI.

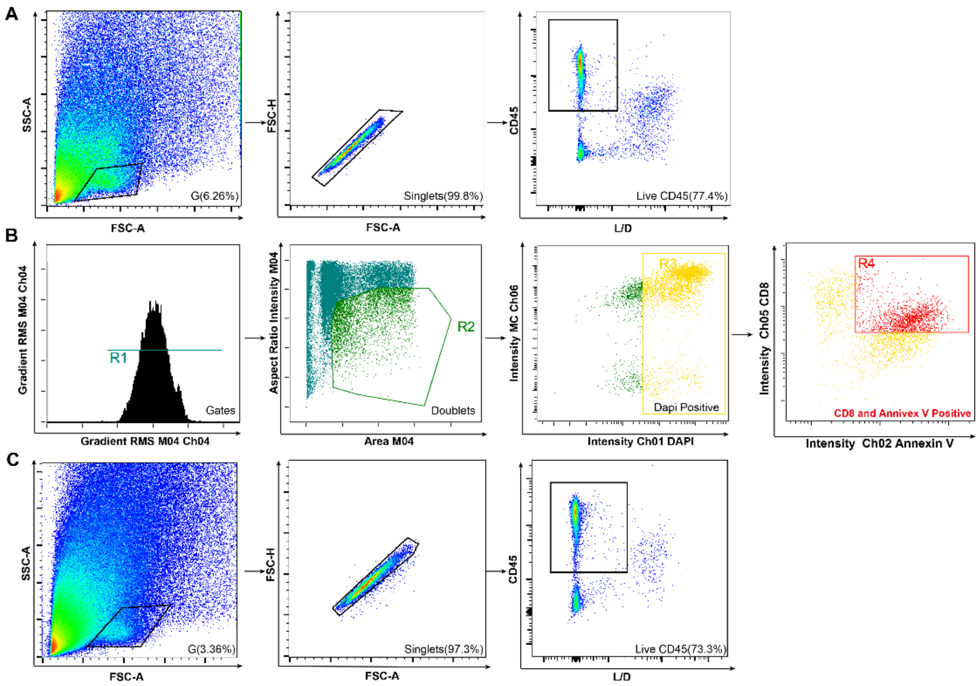

Supplementary Figure 3. **Gating strategy for renal live $CD45^{+}$ cells or $CD8^{+}$, Annexin V^{+}, and $DAPI^{+}$ doublets.**

(a) Gating strategy of single cell suspensions of kidneys from 3 and 14-day uIRI model (FSC, forward scatter; SSC, side scatter; L/D, live or dead.). (b) Gating strategy for selecting doublets in CD8 T and endothelial cells, and then analysis of $CD8^{+}$, Annexin $V^{+,}$ and $DAPI^{+}$ doublets. (c) Gating strategy of single cell suspensions of kidneys from sham, uIRI 14D model treated with CD8α mAb, and the Isotype control group.

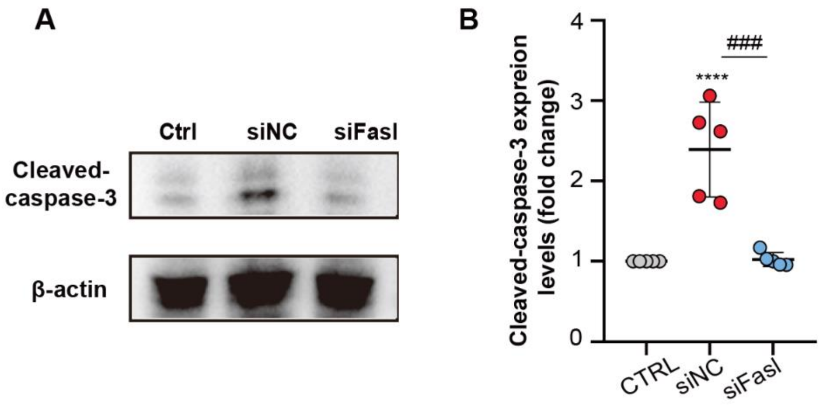

Supplementary Figure 4. **CD8 T cells induced the apoptosis of endothelial cells through Fasl-Fas signaling.**

(a) Western blot analysis was conducted to assess the levels of cleaved-caspase-3 protein in endothelial cells co-cultured with CD8 T cells, transfected with either Fasl siRNA or NC siRNA. (b) The protein levels of cleaved-caspase-3 in sham kidneys of endothelial cells were quantified under similar conditions. Data are presented as means ± SD. **** P <0.0001 compared to control group; ### P <0.001 compared to CD8 T cells transfected with NC siRNA group.

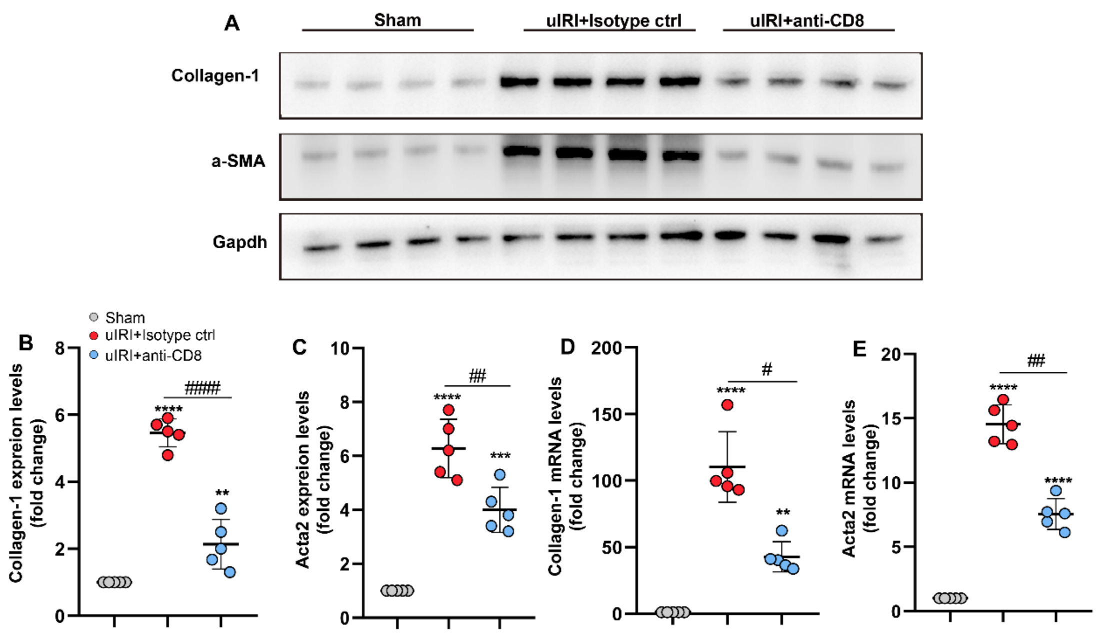

Supplementary Figure 5. **CD8 T cells depletion alleviates renal fibrosis.**

(a)Western blot analysis of Collagen-1 and a-SMA protein levels in the kidneys of sham, uIRI 14D mice treated with Isotype control or anti-CD8α mAb. (b, c) Quantifying the protein levels of Collagen-1 and a-SMA in sham kidneys, uIRI 14D mice treated with Isotype control or anti-CD8α mAb. (d, e) Quantifying the mRNA levels of Acta2 and Collagen-1 in sham kidneys, uIRI 14D mice treated with Isotype control or anti-CD8α mAb. Data are presented as means ± SD. $^{**}P < 0.01$, $^{***}P < 0.001$, $^{****}P < 0.0001$ compared to sham group; $^{\#}P < 0.05$, $^{\#\#}P < 0.01$, $^{\#\#\#\#}P < 0.0001$ compared to uIRI model treated with Isotype Control.
